# L-Histidine Modulates the Catalytic Activity and Conformational Changes of the HD3 Deoxyribozyme

**DOI:** 10.3390/genes15111481

**Published:** 2024-11-17

**Authors:** Nae Sakimoto, Hirofumi Imanaka, Elisa Tomita-Sudo, Tomoka Akita, Junji Kawakami

**Affiliations:** 1 Konan Laboratory for Oligonucleotide Therapeutics (KOLOT), 7-1-20 Minatojima-minamimachi, Chuo-ku, Kobe 650-0047, Hyogo, Japan; 2 Faculty of Frontiers of Innovative Research in Science and Technology, Konan University, 7-1-20 Minatojima-minamimachi, Chuo-ku, Kobe 650-0047, Hyogo, Japan

**Keywords:** deoxyribozyme, l-histidine, catalytic activity, conformational changes, functional nucleic acids, binding constant, circular dichroism, ribonuclease mimicry, small molecule interaction

## Abstract

**Background/Objectives**: Riboswitches are functional nucleic acids that regulate biological processes by interacting with small molecules, such as metabolites, influencing gene expression. Artificial functional nucleic acids, including deoxyribozymes, have been developed through in vitro selection for various catalytic functions. In a previous study, an l-histidine-dependent deoxyribozyme was identified, exhibiting RNA cleavage activity in the presence of l-histidine resembling ribonuclease catalytic mechanisms. This study aims to clarify the role of l-histidine in the activity and structural formation of the l-histidine-dependent deoxyribozyme (HD), focusing on the binding properties and conformational changes of its derivative HD3. **Methods**: Conformational changes in HD3 were analyzed using circular dichroism (CD) under varying concentrations of l-histidine. Direct binding analysis was conducted using carbon-14 (^14^C)-labeled l-histidine and a liquid scintillation counter. The catalytic activity of HD3 in the presence of different l-histidine concentrations was measured. **Results**: The binding constant for l-histidine-induced conformational changes (*K*_a(CD)_) was found to be 2.0 × 10^3^ (M^−1^), whereas for catalytic activity (*K*_a(Rxn)_) and scintillation counting (*K*_a(RI)_), it was approximately 1.0 × 10^3^ (M^−1^). **Conclusions**: l-Histidine plays an essential role in both the catalytic activity and structural formation of the HD3 deoxyribozyme. The consistent binding constants across different experimental methods highlight the significant contribution of l-histidine to the active folding of deoxyribozymes.

## 1. Introduction

Since the discovery of riboswitches, the importance of interactions between RNA and small molecules has received considerable attention. Many natural riboswitches are structurally and functionally altered by small molecules such as metabolites, amino acids, and vitamins [1]. Similar phenomena have been observed for artificial functional RNAs such as aptamers [2,3], allosteric ribozymes [4], and aptazymes [5]. Recently, attempts have been made to utilize functional nucleic acids as therapeutics [6] and biosensing tools [7,8]. Artificial riboswitches and functional nucleic acids have generally been obtained through in vitro selection [9,10,11]. Functional RNAs obtained by in vitro selection have some flexibility regarding the effector molecule and can potentially be used to construct artificial riboswitches with small molecules.

Various functional RNA and DNA molecules have been identified. One of them, an RNA-cleaving deoxyribozyme with l-histidine as a cofactor, was obtained by Roth and Breaker through in vitro selection [12,13]. This concept was inspired by the highly active catalysis of protein enzymes, where ribonucleases utilize the imidazole group of l-histidine as a catalytic residue to achieve the rapid cleavage of nucleic acids. The authors considered the possibility of using amino acids as cofactors to obtain functional nucleic acids that mimic the catalytic mechanism of proteins. The l-histidine-dependent deoxyribozyme (HD) obtained in their studies was found to have a high specificity for l-histidine that was so strong that no other amino acid or d-histidine activated HD. They obtained the pH profile of HD activity [12,14], and the p*K*_a_ of the catalytic residues of HD was similar to that of l-histidine. This suggests that the imidazole group of histidine can be used as a chemical step in the catalytic mechanism. However, in the case of HD2, a derivative of HD, the cleavage activity was found to be *k*_obs_ = 0.2 min^−1^ in the presence of 100 mM of l-histidine, which was high but not up to the activity of the protein enzyme [15]. This does not confirm that the imidazole group of histidine is directly utilized in the chemical step of the cleavage reaction. Several other deoxyribozymes have been identified using similar concepts, but their detailed mechanism remains unclear [16,17]. By clarifying the mechanism by which l-histidine influences HD activity, particularly if the mechanism is the same as that of ribonucleases, re-selection is expected to lead to further improvement in function. Determining the role of l-histidine in HD is important for its widespread application. Quantitative binding analysis was performed to clarify whether HD undergoes a conformational change due to the binding of l-histidine or if it uses l-histidine as a chemical step. Essentially, determining the relationship between the binding constant of l-histidine to HD and its catalytic activity, which has not yet been discussed, is necessary. Therefore, it is crucial to evaluate the binding, concentration-dependent conformational changes, and concentration-dependent changes in catalytic activity in parallel.

In this study, we made a parallel comparison of the l-histidine concentration dependence of the catalytic activity, conformational changes using circular dichroism (CD) [18,19,20] spectra, and direct binding quantification via a liquid scintillation counter using ^14^C-labeled l-histidine.

## 2. Materials and Methods

### 2.1. Functional DNA (Deoxyribozyme)

**Figure 1:**
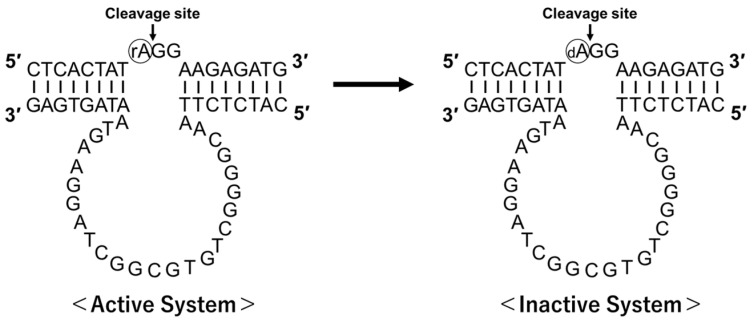
Inactivated histidine-dependent deoxyribozyme for CD measurement. l-Histidine-dependent deoxyribozyme HD3 and its substrates are shown. For the substrate chain used for CD measurements, rA at the cleavage site was changed to dA to prevent cleavage.

HD3, an l-histidine-dependent deoxyribozyme [12], was used for all analyses in this study. DNA and RNA were purchased from Hokkaido System Science (Sapporo, Hokkaido, Japan). DH3 was chemically synthesized and, therefore, histidine contamination should not have occurred. For CD measurements, adenosine at the cleavage site of the substrate RNA was substituted with DNA to prevent cleavage during the analysis. This modification successfully acquired CD spectra at various l-histidine concentrations under the condition that the active structure could be formed but the cleavage reaction did not occur (Figure 1).

### 2.2. Kinetic Analysis of the RNA Cleavage Reaction

Pseudo-first-order kinetic parameters were determined by fixing the substrate strand at 30 nM, changing the concentration of the enzyme strand (HD3) (3.0, 6.0, 12.0, and 18.0 µM), and analyzing the change in the amount of cleavage product per time. Reactions were performed under buffer conditions containing 10 mM of l-histidine, 50 mM of HEPES (pH 7.5 at 25 °C), 500 mM of NaCl, 500 mM of KCl, and 0.5 mM of EDTA. After the reaction, a 2× volume of the stop solution (50% *v*/*v* formamide/50 mM phosphate buffer (pH 8.0 at 25 °C)) was added, and the reaction was stopped by heating at 94 °C for 2 min and quickly chilling on ice for denaturation. RNA was separated using 7 M of urea denaturing PAGE and stained using SYBR Gold (Thermo Fisher Scientific K.K., Tokyo, Japan). Fluorescence images were captured using an O580 filter and a SHG532 laser on an image analyzer (FLA5100; FUJIFILM, Tokyo, Japan) and visualized using Multi Gauge V4.21. The fluorescence intensities of the uncleaved and cleaved RNA were quantified, and the cleaved RNA product yield (product yield) was calculated for each time point using Equation (1).

Product yield (%) = 100 × RNA cleaved/(RNA uncleaved + RNA cleaved)(1)

The observed reaction rate constant (*k*_obs_) was determined from the change in the product yield over time. The time course of the product yield was fitted to Equation (2) to determine the rate constant using curve-fitting software (Win Curve Fit Ver. 1.0), where, b, t, and c represent the maximum cleavage ratio, time, and correction values, respectively.

Product yield = b{1 − exp^(−*kobs*·t)^} + c (2)

### 2.3. Analysis of the Binding Constant Using CD Spectra

The l-histidine concentration dependency of the HD3 conformational change was evaluated via CD spectroscopy using J-820 (JASCO, Tokyo, Japan). A DNA solution (200 µL of 30 µM DNA) in 50 mM of HEPES (pH 7.5), 500 mM of NaCl, 500 mM of KCl, and 0.5 mM of EDTA at 25 °C with 0, 0.1, 0.2, 0.3, 0.4, 0.5, 0.75, 1.0, 2.0, 5.0, and 10 mM of cofactors (l-histidine, l-arginine, and Mg^2+^) was used for the CD measurement. From the change in CD intensity at 267 nm due to l-histidine binding, the binding constants of HD3 and l-histidine (*K*_a(CD)_) were determined through a curve-fitting method using Equation (3), where ∆θ, ∆θ_max_, [D]_0_, and [His]_0_ denote the change in molar ellipticity at 267 nm and the maximum change in θ, as well as the initial concentrations of deoxyribozyme and histidine, respectively.

∆θ = ∆θ_max_ {1 + *K*_a_[D]_0_ + *K*_a_[His]_0_ − [(1 + *K*_a_[D]_0_ + *K*_a_[His]_0_)^2^ − 4·*K*_a_^2^[D]_0_[His]_0_]^1/2^}/2·*K*_a_[D]_0_ (3)

### 2.4. Calculation of Binding Constants by Column Adsorption Experiments

**Figure 2:**
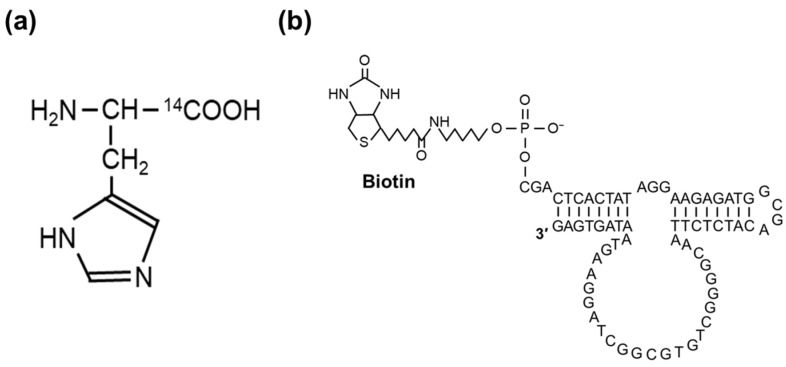
Chemical structures of (**a**) ^14^C-labeled l-histidine and (**b**) biotinyl-HD3-2.

All substrate strands converted to DNA were linked to HD3 by a stable hairpin loop (GCGA), and a DNA strand with a biotinyl modification at the 5′ end was designed (Biotinyl-HD3-2, Figure 2). The biotinyl-HD3-2 solution was mixed with avidin-attached agarose beads (Funakoshi Co., Ltd., Tokyo, Japan) to immobilize the deoxyribozyme on the surface of the resin. ^14^C-labeled l-histidine was purchased from the Japan Radioisotope Association (Tokyo, Japan). The ^14^C-labeled l-histidine was dissolved in 50 mM of HEPES buffer (pH 7.5 at 25 °C) containing 500 mM of NaCl, 500 mM of KCl, and 0.1 mM of EDTA at varied concentrations up to 50 mM. The solutions were incubated with the DNA-immobilized resin at 25 °C for 1 h, and the amount of bound histidine (^14^C) was quantified in a liquid scintillation counter (Aloka, Japan).

The measured ΔSI (change in scintillation intensity) was used for the *K*_a(RI)_ calculation according to the following Equation (4), where ΔSI_max_ denotes the maximum change in ΔSI.

ΔSI = ΔSI_max_ {1 + *K*_a_[D]_0_ + *K*_a_[His]_0_ − [(1 + *K*_a_[D]_0_ + *K*_a_[His]_0_)^2^ − 4·*K*_a_^2^[D]_0_[His]_0_]^1/2^}/2·*K*_a_[D]_0_ (4)

## 3. Results

### 3.1. Histidine Concentration Dependency for HD3 Activity

**Figure 3:**
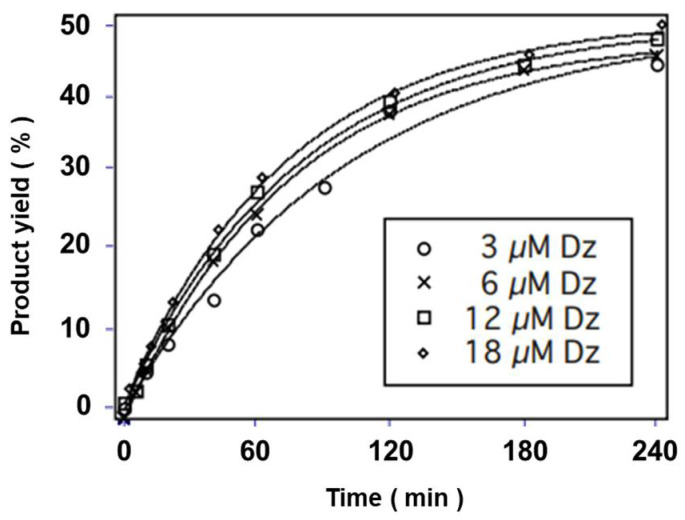
Kinetic analysis of the RNA cleavage reaction by HD3. The experiments were performed in 50 mM of HEPES buffer (pH 7.5 at 25 °C) containing 500 mM of NaCl, 500 mM of KCl, and 0.5 mM of EDTA at 25 °C.

Hundred- to thousand-fold higher amounts of HD3 were used against 30 nM substrate strands. No considerable change in the cleavage reaction was observed at HD3 concentrations of 6 µM or higher in the presence of 10 mM of l-histidine (Figure 3). This confirms that the pseudo-first-order reaction was established with 6 µM or more of HD3. The reaction rate constants for each HD3 concentration are listed in Table 1.

Next, the l-histidine concentration was varied (2.0, 10, and 50 mM) with a fixed HD3 concentration at 6 µM, and the pseudo-first-order rate constant was measured. The correlation between the logarithm of the reaction rate constant and the logarithm of the l-histidine concentration on the abscissa showed that the activity increased as the concentration changed (Supplementary Figure S1). However, in the l-histidine concentration range above 10 mM, the apparent rate constant change with l-histidine concentration did not exhibit a slope of 1. This suggests that the dissociation constant is even lower. This observation approximates the activity profile of HD3 previously reported by Breaker et al. The apparent binding constant (*K*_a(Rxn)_) for HD3, predicted from the results obtained in this study, was expected to be 1 × 10^3^ M^−1^.

### 3.2. Analysis of CD Spectra

We chose to utilize CD spectra to evaluate conformational changes in nucleic acids. Currently, X-ray crystallography [21], NMR, and cryo-EM [22] are the main methods used for the structural analysis of nucleic acids. However, these methods are time consuming and require large amounts of nucleic acid for analysis. In addition, X-ray and cryo-EM analyses are static; thus, these methods are not suitable for the quantification of the slight structural changes in large DNA molecules resulting from small amino acid binding. In contrast, CD does not require high sample concentrations, and the analysis time can be substantially shortened. Therefore, we considered CD to be an appropriate method for simple and quick evaluation of conformational changes.

**Figure 4:**
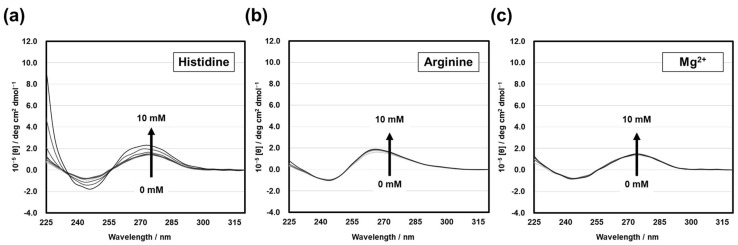
CD spectra of the substrate-deoxyribozyme (HD3) complex with (**a**) l-histidine, (**b**) l-arginine, and (**c**) Mg^2+^. DNA samples were prepared at a concentration of 30 µM in 50 mM of HEPES buffer (pH 7.5 at 25 °C) containing 500 mM of NaCl, 500 mM of KCl, and 0.5 mM of EDTA. Measurements were performed using cofactor concentrations of 0, 0.1, 0.2, 0.3, 0.4, 0.5, 0.75, 1.0, 2.0, 5.0, and 10 mM.

To measure the CD spectra, only one oxygen atom of the substrate at the cleavage site was eliminated so that an active structure could be formed; however, the cleavage reaction by HD3 was not allowed to occur. CD spectra were measured when the l-histidine concentration was varied from 0 mM to 10 mM in the presence of 30 µM of HD3 at pH 7.5 (Figure 4a). The signal intensity of the positive peak near 270 nm and the negative peak near 245 nm increased with the addition of l-histidine, indicating that a right-handed spiral structure was induced by l-histidine. This change was considered to be a two-state transition because of the isodichroic point at approximately 255 nm.

To confirm whether this spectral change at 270 nm was due to an l-histidine–induced conformational change in HD3, we examined spectral changes in the presence of l-arginine and magnesium ions. No significant changes in the CD patterns were observed in either case (Figure 4b,c). Previous studies have shown that HD3 is inactive in the presence of l-arginine and magnesium ions [12]. Therefore, the significant CD change at 270 nm observed only in the presence of l-histidine is considered to be due to the formation of structures essential for catalytic activity.

**Figure 5:**
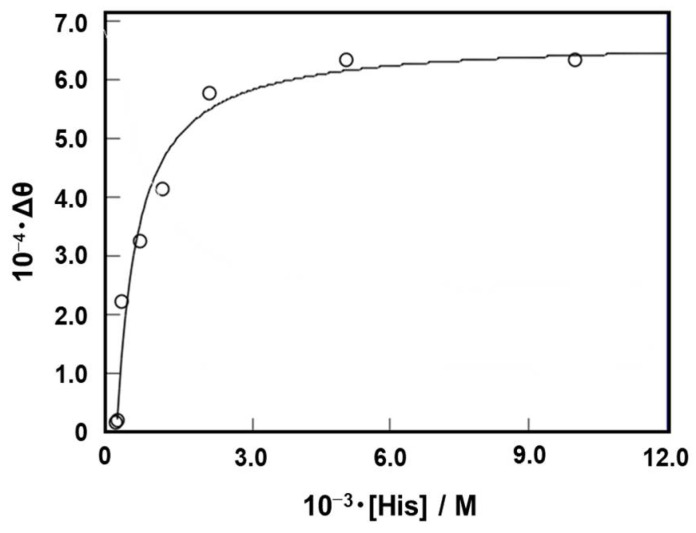
Titration curves of CD intensity at 267 nm as a function of l-histidine concentration. The binding constant between the deoxyribozyme and l-histidine was determined using a curve-fitting procedure with the following equation: ∆θ = ∆θ_max_ {1 + *K*_a_[D]_0_ + *K*_a_[His]_0_ − [(1 + *K*_a_[D]_0_ + *K*_a_[His]_0_)^2^ − 4·*K*_a_^2^[D]_0_[His]_0_]^1/2^}/2·*K*_a_[D]_0_, where θ denotes the molar ellipticity at 267 nm, and θ_max_, [D]_0_, and [His]_0_ are the maximum change in θ, initial concentration of DNA, and initial concentration of l-histidine, respectively.

As shown in Figure 4a, the binding between HD3 and l-histidine was quantified from the change in signal intensity at a wavelength of 267 nm. The binding constant (*K*_a(CD)_) was calculated by curve fitting to be approximately 2.0 × 10^3^ M^−1^ (Figure 5). However, the binding observed here may reflect histidine binding, which induces a conformational change, and may not assess the critical binding of l-histidine for catalytic activity. Therefore, an HD3-immobilized column was employed, and the binding of HD3 to ^14^C-labeled l-histidine was measured using a liquid scintillation counter.

### 3.3. Determination of Binding Constant Between HD3-2 and ^14^C-Labeled l-Histidine

Liquid scintillation counters are used to measure low-energy β-rays such as those emitted by ^14^C. By using a liquid scintillator that emits fluorescence when exposed to radiation, the excited fluorescence can be measured to provide sensitive and quantitative measurements of the labeled substances. In this study, HD was fixed in a column, and ^14^C-labeled l-histidine was applied to the column to quantify the binding of l-histidine. After column adsorption, the radioactivity on the column was evaluated using a liquid scintillation counter.

**Figure 6:**
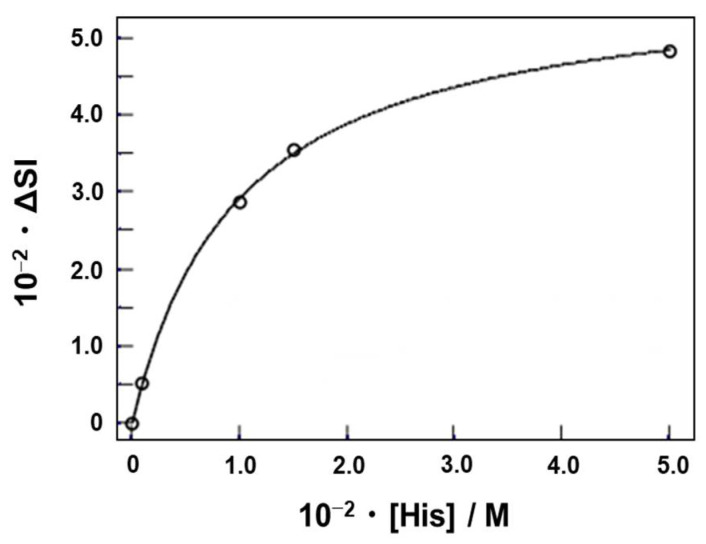
Concentration dependency profile and curve fitting of histidine binding to HD3-2. Binding experiments were performed in 50 mM of HEPES buffer (pH 7.5 at 25 °C) containing 500 mM of NaCl, 500 mM of KCl, and 0.1 mM of EDTA. Cells were incubated for 1 h at 25 °C.

For this analysis, HD3-2 was used, in which the substrate sequence and enzyme chains were linked through a stable hairpin loop (Figure 2). The ^14^C-labeled l-histidine was incubated with the HD3-2-immobilized resin for 1 h at 25 °C, and the binding amount was quantified (Figure 6). As a result, stoichiometry could be analyzed at 1:1, and its binding constant (*K*_a(RI)_) was calculated to be approximately 1.0 × 10^3^ M^−1^. This result was similar to the CD results. This suggests that the conformational change caused by histidine observed in CD is necessary for catalytic activity.

## 4. Discussion

Various functional nucleic acids have been obtained through in vitro selection, including RNA-cleaving deoxyribozymes that use l-histidine as a cofactor [12]. HD deoxyribozyme was found to have high specificity for l-histidine, while other amino acids and d-histidine did not. In a report by Breaker et al., the pH profile of HD activity was analyzed, and the results showed that the p*K*_a_ of the catalytic residue was similar to that of l-histidine. Their findings suggested that the imidazole group of histidine may serve as a chemical step in the catalytic mechanism. These results also indicate the feasibility of developing a deoxyribozyme with activity comparable to that of protein enzymes. In this study, we clarified and evaluated the role of l-histidine in HD, focusing on the binding constants of l-histidine to HD and its catalytic capacity. The binding constants of l-histidine to HD were assessed based on the structural changes in the CD spectra and direct binding analysis of radioactive isotope scintillation counting (RI) with ^14^C-labeled l-histidine.

In summary, the dependence of catalytic activity on l-histidine concentration observed in this study is consistent with the activity profile report by Breaker et al., which indicated an apparent binding constant of *K*_a(Rxn)_ ≈ 1 × 10^3^ M^−1^ [12]. Next, the binding constant was calculated from the change in the CD spectrum as *K*_a(CD)_ = 2.0 × 10^3^ M^−1^, and the binding constant obtained from the column adsorption experiment using ^14^C-labeled l-histidine was calculated as *K*_a(RI)_ = 1.0 × 10^3^ M^−1^. The RI results show that the binding ratio of l-histidine to HD3 was 1:1. The p*K*_a_ of the catalytic residue was approximately 6.0 based on the pH profile obtained in a previous study [12,14], which is consistent with the p*K*_a_ of the imidazole group of histidine. This suggests that l-histidine acts as a catalytic residue in the cleavage reaction mechanism. However, our CD results demonstrate that l-histidine is crucial for forming the active structure, as significant structural changes occur only in its presence. While these findings do not entirely exclude l-histidine as a catalytic residue, they strongly suggest its role in facilitating active enzymatic folding. Owing to its high specificity for l-histidine, HD3 is expected to regulate gene expression via amino acid metabolism. For example, mutation of arginine to histidine is frequently observed in cancer cells and is associated with increased cell proliferation in fibroblasts [23]. This suggests that histidine uptake is higher in cancer cells than in normal cells and that HD may have potential applications as a new regulatory tool for targeting mRNAs expressed in cancer cells. Moreover, HD3 has been used as a biosensor [8] and is expected to serve as an important tool in biological research [24].

HD3 is expected to be utilized as an aptazyme because it has a very high histidine discrimination ability and expresses cleavage activity by interacting with histidine. However, there are specific limitations to be addressed in using HD3 as a biotechnological tool. For cleavage activity, HD3 has a smaller kinetic constant than that of 10–23 deoxyribozyme [25]. However, previous studies on gene expression regulation by FANAzyme (in which FANA-modified nucleic acids were introduced into deoxyribozyme [26]) and modified deoxyribozyme (in which LNA was introduced into 8–17 deoxyribozyme [27]) showed that the activity of both deoxyribozymes was comparable to that of HD3. Therefore, cleavage activity does not seem to be a problem for intracellular application. More importantly, nuclease resistance is acquired by introducing a modified nucleic acid. Considering intracellular applications, modified nucleic acids should be introduced into sequences other than the catalytic core of HD3 to provide nuclease resistance.

The next issue is to increase the binding affinity of histidine. The dissociation constant (*K*_d_) of conventional nucleic acid aptamers is at a nanomolar scale. Macgen, a nucleic acid aptamer that binds to vascular endothelial growth factor (VEFG), is certified as a nucleic acid drug for the treatment of age-related macular degeneration [28,29,30,31]. In contrast, the *K*_d_ for HD3 is 1 mM, likely because HD3 targets amino acids rather than proteins, which limits the interaction points. Since instances have been reported where the binding affinity of DNA aptamers was improved by populated bases [32], further clarification of the interaction mechanism of HD3 with histidine may effectively improve the binding affinity. For example, HD3 with improved binding affinity to histidine, as well as with modified nucleic acids in the target gene-binding sequence for improved binding affinity and nuclease resistance, may serve as a tissue-specific biotool for cancer cells that take up a lot of histidine.

## 5. Conclusions

To understand the role of histidine in HD3, the binding constants calculated from the catalytic activity of HD3 were compared to those of the conformational changes. The binding constants calculated from the CD spectra were consistent with those obtained from direct binding observations using ^14^C. They also aligned with the binding constants inferred from the l-histidine concentration-dependent activity profiles. This indicates that l-histidine contributes to the formation of the active structure, although its function as a catalytic residue cannot be completely ruled out. These results provide new insights into the HD3 mechanism by comparing catalytic activity and structural changes in parallel. 

## Figures and Tables

**Table 1:** Pseudo-first-order kinetic parameters ^1^.

HD3 Concentration (μM)	Maximum Cleavage Ratio (%)	*k*_obs_ (10^−2^ min^−1^)
3.0	51 ± 3.3	1.0 ± 0.1
6.0	50 ± 0.4	1.3 ± 0.1
12.0	48 ± 0.8	1.4 ± 0.1
18.0	50 ± 1.0	1.3 ± 0.1

^1^ Cleavage reactions were carried out with 30 nM of the substrate and 10 mM of l-histidine in 50 mM of HEPES buffer (pH 7.5 at 25 °C) containing 500 mM of NaCl, 500 mM of KCl, and 0.5 mM of EDTA.

## Data Availability

All data generated or analyzed during this study are available from the corresponding author on reasonable request.

## Supplementary Materials

The following supporting information can be downloaded at https://www.mdpi.com/article/10.3390/genes15111481/s1, Figure S1: Kinetic analysis of the RNA cleavage reaction by HD3 using the log *k*_obs_ vs. log [His] plot.
