# Supplementary material for: L-Histidine Modulates the Catalytic Activity and Conformational Changes of the HD3 Deoxyribozyme
Source: Genes (Basel). 2024 Nov 17;15(11):1481. doi: 10.3390/genes15111481 (PMC11594175; doi:10.3390/genes15111481)
Supplement: Supplementary file 1 [file genes-15-01481-s001.zip › genes-3293255-supplementary.pdf]

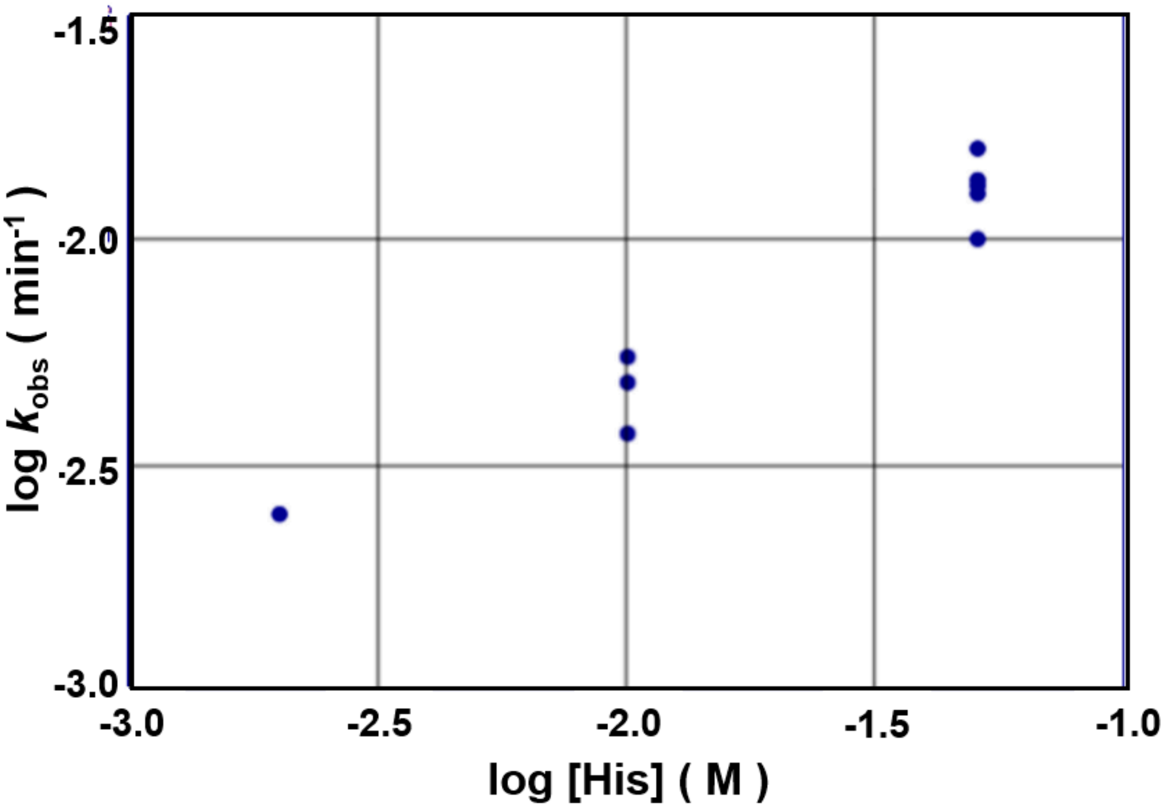

Supplementary Figure S1. Kinetic analysis of the RNA cleavage reaction by HD3 using the log k_{obs} vs. log [His] plot. Measurements were done in 50 mM HEPES buffer (pH7.5 at 25˚C) containing 500 mM NaCl, 500 mM KCl, and 0.5 mM EDTA.
